# The Efficacy of Synchronous Combination of Chemotherapy and EGFR TKIs for the First-Line Treatment of NSCLC: A Systematic Analysis

**DOI:** 10.1371/journal.pone.0135829

**Published:** 2015-08-18

**Authors:** Han Yan, Huihui Li, Qin Li, Pengfei Zhao, Wei Wang, Bangwei Cao

**Affiliations:** 1 Department of Oncology, Beijing Friendship Hospital, Capital Medical University, Beijing, China; 2 Medical Healthcare Center, Beijing Friendship Hospital, Capital Medical University, Beijing, China; University of North Carolina School of Medicine, UNITED STATES

## Abstract

**Background:**

The combination of chemotherapy and epidermal growth factor receptor (EGFR) tyrosine kinase inhibitors (TKIs) currently has become the hotspot issue in the treatment of non-small lung cancer (NSCLC). This systematic review was conducted to compare the efficacy and safety of the synchronous combination of these two treatments with EGFR TKIs or chemotherapy alone in advanced NSCLC.

**Methods:**

EMBASE, PubMed, the Central Registry of Controlled Trials in the Cochrane Library (CENTRAL), Chinese biomedical literature database (CNKI) and meeting summaries were searched. The Phase II/III randomized controlled trials were selected by which patients with advanced NSCLC were randomized to receive a combination of EGFR TKIs and chemotherapy by synchronous mode vs. EGFR TKIs or chemotherapy alone.

**Results:**

A total of six randomized controlled trials (RCTs) including 4675 patients were enrolled in the systematic review. The meta-analysis demonstrated that the synchronous combination group of chemotherapy and EGFR TKIs did not reach satisfactory results; there was no significant difference in overall survival (OS), time to progression (TTP) and objective response rate (ORR), compared with monotherapy (OS: HR = 1.05, 95%CI = 0.98–1.12; TTP: HR = 0.94, 95%CI = 0.89–1.00; ORR: RR = 1.07, 95%CI = 0.98–1.17), and no significant difference in OS and progression-free survival (PFS), compared with EGFR TKIs alone (OS: HR = 1.10, 95% CI = 0.83–1.46; PFS: HR = 0.86, 95% CI = 0.67–1.10). The patients who received synchronous combined therapy presented with increased incidences of grade 3/4 anemia (RR = 1.40, 95% CI = 1.10–1.79) and rash (RR = 7.43, 95% CI = 4.56–12.09), compared with chemotherapy, grade 3/4 anemia (RR = 6.71, 95% CI = 1.25–35.93) and fatigue (RR = 9.60, 95% CI = 2.28–40.86) compared with EGFR TKI monotherapy.

**Conclusions:**

The synchronous combination of chemotherapy and TKIs is not superior to chemotherapy or EGFR TKIs alone for the first-line treatment of NSCLC.

## Introduction

According to the International Epidemiology, the incidence and mortality rates of lung cancer are located in the top three of all malignancies. NSCLC accounts for 80% of lung cancer. Despite the great progress that has been achieved in surgery, radiotherapy and chemotherapy, advanced NSCLC still has a very low five-year survival rate.

Platinum-based combination chemotherapy is the first-line therapy for advanced NSCLC. In recent years, the application of EGFR TKIs, such as gefitinib and erlotinib, provided a new approach for the treatment of NSCLC. The Iressa Pan-Asia Study (IPASS) study showed that gefitinib had high efficacy in lung adenocarcinoma patients with EGFR mutations [1]. At the same time, multiple studies confirmed that the selective application of gefitinib was relatively equally effective with chemotherapy in the first-line treatment of NSCLC [2,3]. However, the efficacy of chemotherapy and EGFR TKIs has recently reached a plateau. Currently, combination therapy with chemotherapy and TKIs has become the hotspot. The combination of chemotherapy and TKI has two modes: the interleaved mode, where chemotherapy and TKI are administered in a certain sequential order, and synchronous mode, where chemotherapy and TKI are given at the same time. Preclinical studies have exhibited that erlotinb showed additive or synergism effect with chemotherapy [4]. A Phase III multi-center clinical trial showed that gefitinib in combination with gemcitabine and cisplatin by the synchronous mode did not provide a survival benefit, compared with chemotherapy alone [5]. Another phase III study also achieved similar results [6]. There are two possible reasons: first, chemotherapy combined with TKI synchronously may have antagonistic effects; second, the patients had not been selected according to EGFR status. However, what about the results of chemotherapy combined with TKI by synchronous mode in the patients with EGFR mutations? Herbst showed that the OS in the two treatment modalities was no different in patients with EGFR mutations or wild-type EGFR [7]. Perhaps larger clinical trials are needed to obtain positive results. There are several clinical trials have been conducted for chemotherapy combined with EGFR TKIs vs. EGFR TKI monotherapy in advanced NSCLC [8,9]. The 30406 Trial demonstrated that erlotinib combined with chemotherapy had a similar effect, compared with erlotinib alone for the treatment of clinically selected patients with advanced NSCLC[8], whereas another clinical trial reported that a combination of gefitinib and chemotherapy had better PFS than gefitinib alone [9]. Whether a combination of EGFR TKIs and chemotherapy by synchronous mode is superior to EGFR TKIs or chemotherapy alone in advanced NSCLC remains controversial. Thus, we performed a systematic review to compare the efficacy and safety of the synchronous combination of the two treatments with EGFR TKIs or chemotherapy alone in advanced NSCLC.

## Patients and Methods

### Search Method

EMBASE (1974 to January 2015), PubMed (1966 to January 2015), the CENTAL database, European Society for Medical Oncology (ESMO), the annual meetings of the American Society of Clinical Oncology (ASCO) and CNKI were searched. The medical subjects heading (MeSH) terms included: lung neoplasms, pulmonary neoplasm, lung neoplasm, pulmonary neoplasm, lung cancers, lung cancer, pulmonary cancer and pulmonary cancers.

### Inclusion criteria

First, Phase II/III RCTs where the primary endpoint of the clinical trial was OS or PFS were selected; then, patients with pathologically diagnosed NSCLC who were randomized to receive a combination of EGFR TKIs and chemotherapy by synchronous mode vs. EGFR TKIs or chemotherapy alone as the first-line treatment were selected. Only the most recent literature was chosen, if there were multiple articles reported based on the same clinical trials. Nonrandomized studies, ongoing clinical trials and studies without survival data were excluded. If there were no adequate data in the RCTs, the reviewer (Han Yan or Qin Li) will attempt to contact the authors to acquire those missing data.

### Data extraction

Two reviewers (Han Yan and Qin Li) independently searched the literature and read the titles, abstracts or full texts of the literature to determine whether to include the document. Cases of disagreement were resolved by discussion or determined by the third reviewers (Huihui Li). The following information needed to be extracted from the literature: publication year, journal name, the author's name, patients’ race, diseases, methods of randomization, objective response rate (ORR), OS, PFS and Time-to-Progression (TTP) and grades 3 to 4 adverse events (AEs). The quality of the inclusive RCTs was evaluated according to the Cochrane Handbook 4.2.6 for Systematic Reviews of Interventions [10].

### Statistical Analysis

Stata version 12.0 software (Stata Corporation, College Station, Texas, USA) was used to conduct the systematic review. Hazard ratio (HR) and its 95% confidence interval (CI) were collected to estimate the overall effect of PFS and OS. If HR>1.0, it indicates that there is more progression or death in the combination group. The odds ratio (OR) was used to estimated ORR and grade 3 or 4 AEs. If OR>1.0, it indicates that ORR or the incidence of grade 3 or 4 AEs is higher in the combination group. In each systematic review, the Cochran’s χ^2^ test was used to evaluate the heterogeneity of the included clinical trials. The random-effects model (REM) was adopted when I^2^>50, and the heterogeneity could not be explained; otherwise, the fixed-effects model (FEM) was used. The Begg’s test and Egger’s test were adopted to detect any publication bias.

## Results

### Data characteristics and quality assessment

**Fig 1:**
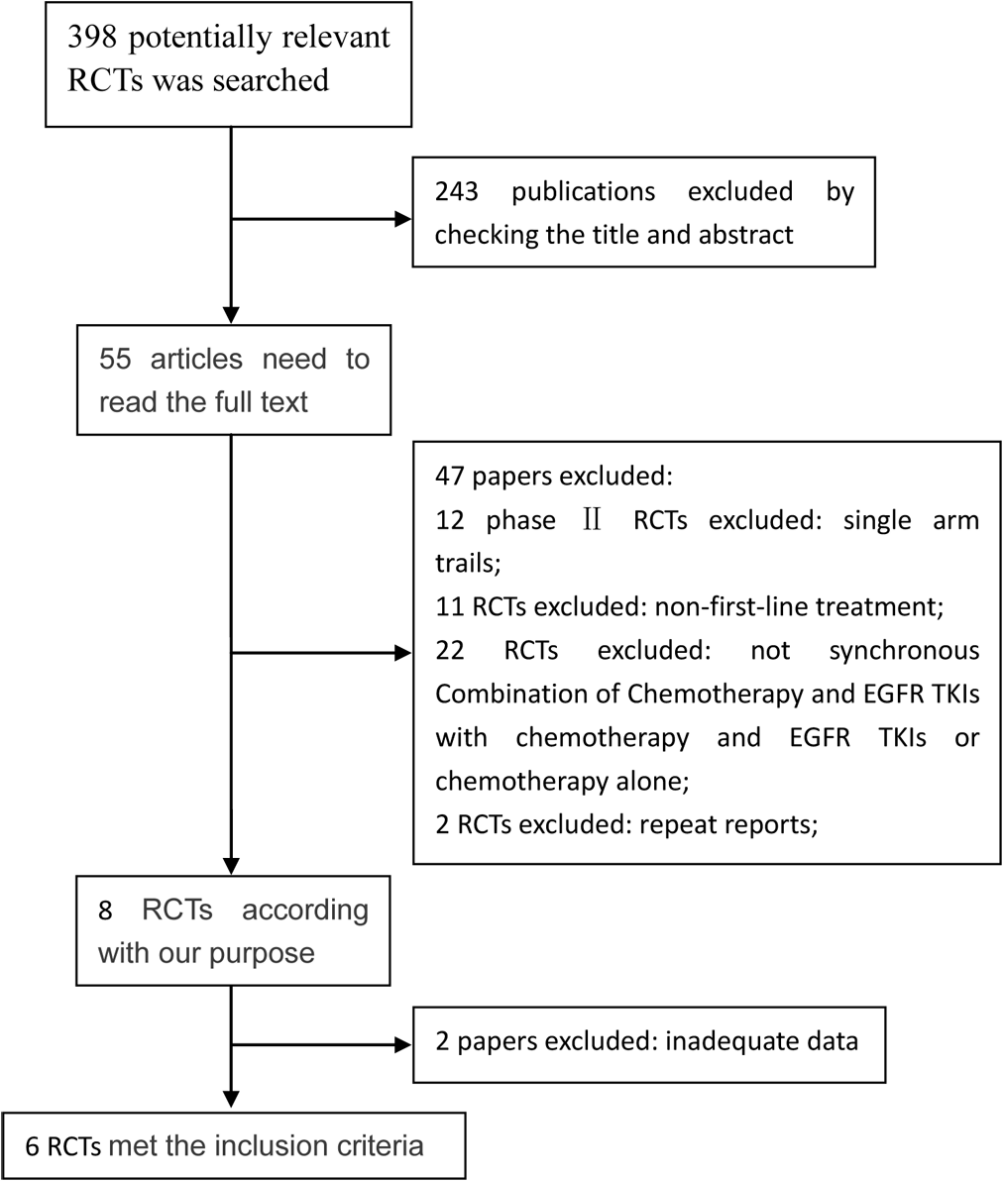
Flow chart of trial selection process.

The detailed steps of the systematic review are shown in Fig 1, as Preferred Reporting Items for Systematic Review and Meta-analyses (PRISMA) flow chart. Six clinical trials including 4675 patients were enrolled in this systematic review [5–8,11,12]. There were 2679, 1864 and 132 patients who were randomized to receive chemotherapy concurrently with EGFR TKI, chemotherapy or EGFR TKI alone, respectively. The studies’ characteristics are shown in Table 1. Five studies compared combination therapy with chemotherapy alone, and two studies compared combination therapy with EGFR TKI monotherapy, and one study compared the efficacy between the three groups. In the six studies, the chemotherapy regimens included gemcitabine/ cisplatin, paclitaxel/carboplatin, and gemcitabine alone, whereas the EGFR TKIs applied in the six studies were gefitinib and erlotinib.

The methodological quality of the included studies was independently assessed by two authors following the Cochrane Handbook for Systematic Reviews of Interventions[10]. The Cochrane Collaboration's tool for assessing the risk of bias was adopted to assess the bias of the six studies. The details of the assessment are shown in S1 Fig.

**Table 1:** Characteristics of included studies.

Clinical Trials	year	phase	country	group	Primary endpoint	No. of patients	CR+PR (%)	OS (m)	PFS (m)	TTP (m)
TALENT: Gatzemeier et al.	2007	Ⅲ	Europe, Canada, South America, and Australasia	erlotinib 150 mg/d daily, gemcitabine 1,250 mg/m^2^ D1 and D8, cisplatin 80 mg/m^2^ D1	OS	580	31.5	10.3	NE	5.7
				gemcitabine 1,250 mg/m^2^ D1 and 8 and cisplatin 80 mg/m^2^ D1		579	29.9	10	NE	5.5
NTACT 1: Giaccone et al.	2004	Ⅲ	European, America, Asia, South Africa	cisplatin 80 mg/m^2^ D1, gemcitabine 1,250 mg/m^2^ D1 and D8, gefitinib 500 mg/d daily	OS	365	49.7	9.9	5.5	NE
				cisplatin 80 mg/m^2^ D1, gemcitabine 1,250 mg/m^2^ D1 and D8, gefitinib 250 mg/d daily		365	50.3	9.9	5.8	NE
				cisplatin 80 mg/m^2^ D1, gemcitabine 1,250 mg/m^2^ D1 and D8, placebo daily		363	44.8	109	6	NE
INTACT 2: Herbst et al.	2004	Ⅲ	United States	paclitaxel 225 mg/m^2^ D1, carboplatin AUC 6 D1, gefitinib 500 mg/ d daily	OS	347	30	8.7	NE	4.6
				paclitaxel 225 mg/m^2^ D1, carboplatin AUC 6 D1, gefitinib 250 mg/ d daily		345	30.4	9.8	NE	5.3
				paclitaxel 225 mg/m^2^ D1, carboplatin AUC 6 D1		345	28.7	9.9	NE	5
TRIBUTE: Herbst et al.	2005	Ⅲ	Global	paclitaxel 200mg/m^2^ D1, carboplatin AUC 6 D1, erlotinib 150mg/day daily	OS	526	21.5	11	NE	6.6
				paclitaxel 200mg/m^2^ D1, carboplatin AUC 6 D1		533	19.3	11	NE	4.3
Stinchcombe et al.	2011	Ⅱ	United States	gemcitabine 1000 mg/m^2^ D1 and D8, erlotinib 100 mg/d daily	NE	51	21	5.6	4.1	NE
				gemcitabine 1000 mg/m^2^ D1		44	7	6.8	3.7	NE
				erlotinib 150 mg/d daily		51	0	5.8	2.8	NE
CALGB 30406: Jänne et al.	2012	Ⅱ	Global	paclitaxel 200mg/m^2^ D1, carboplatin AUC 6 D1,erlotinib 150 mg/d daily	PFS	100	46	20	6.6	NE
				erlotinib 150 mg/d daily		81	35	25	5	NE

This systematic review was performed in accordance with the guidelines of the PRISMA statement [11](see S1 Table).

### Synchronous combination of chemotherapy and TKIs vs. chemotherapy alone

**Fig 2:**
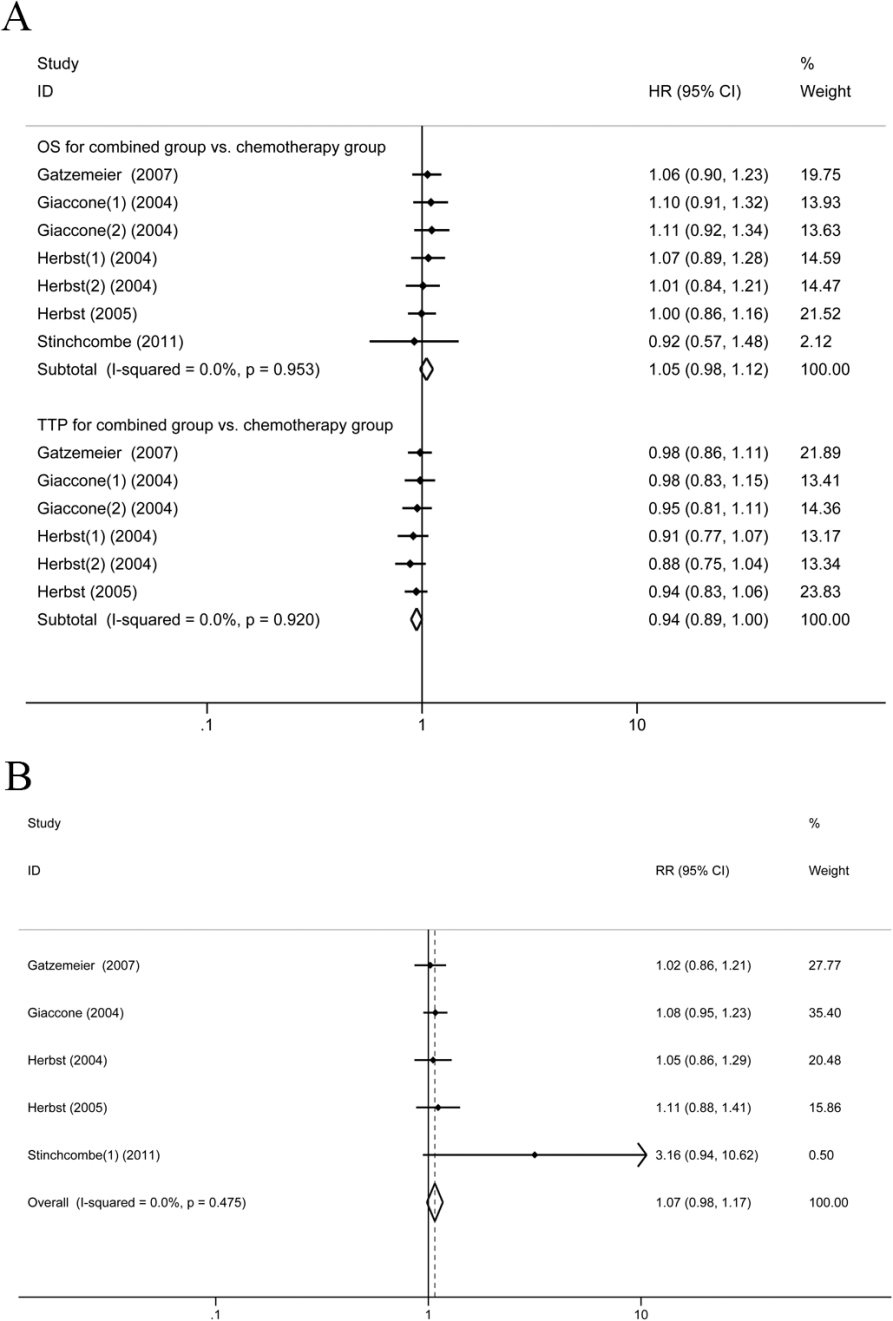
Synchronous combination group vs. chemotherapy (A) OS and TTP for. (B) ORR.

Five trials [5–7,12,13] reported the data on OS comparing the synchronous combination of chemotherapy and TKIs vs. chemotherapy alone. There was no statistical heterogeneity in these studies; therefore, the FEM was applied. The systematic review showed that there was no significant difference in OS between the synchronous combination group and chemotherapy group (HR1.05, 95%CI: 0.98–1.12, *P* = 0.18) (Fig 2A). In five studies, only one study [13] reported the PFS; in the synchronous combination group and chemotherapy group, there was no significant difference between the two groups (HR = 0.77, 95%CI: 0.51–1.17, *P* = 0.217). In the five studies, four studies reported TTP in the synchronous combination therapy group vs. the monotherapy group. FEM was applied for heterogeneity between studies and was low (I^2^ = 0%). The pooled HR was 0.94 and 95% CI is 0.89 to 1.00 indicating a similar TTP in the two groups (*P* = 0.054). Five trials [5–7,12,13] assessed ORR and found no significant advantage of combination therapy over chemotherapy (RR = 1.07, 95%CI: 0.98–1.17, *P* = 0.112) (Fig 2B).

**Fig 3:**
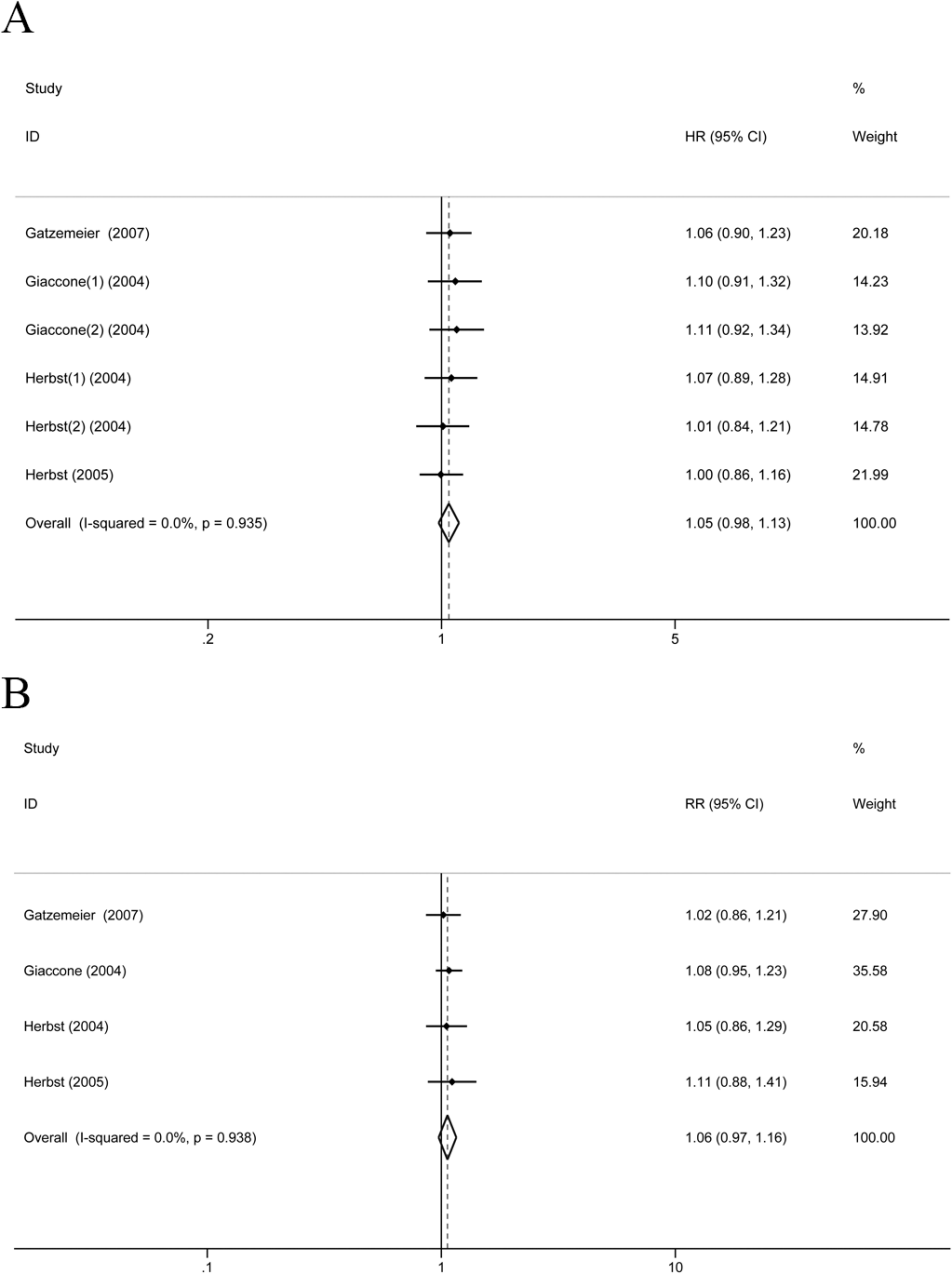
Platinum-containing chemotherapy regimens combined with EGFR TKIs vs. chemotherapy group (A) OS. (B) ORR.

Four studies reported OS and ORR of the platinum-containing chemotherapy combined with EGFR TKIs vs. chemotherapy alone. FEM was applied for the heterogeneity between studies and was low (I^2^ = 0%). The systematic review showed that there was no significant difference in OS and ORR between the two groups (OS: HR = 1.05, 95%CI: 0.98–1.13, *P* = 1.60; ORR:RR = 1.06, 95%CI: 0.97–1.16, P = 0.173) (Fig 3A and 3B).

### Synchronous combination of chemotherapy and TKIs vs. EGFR TKI alone

**Fig 4:**
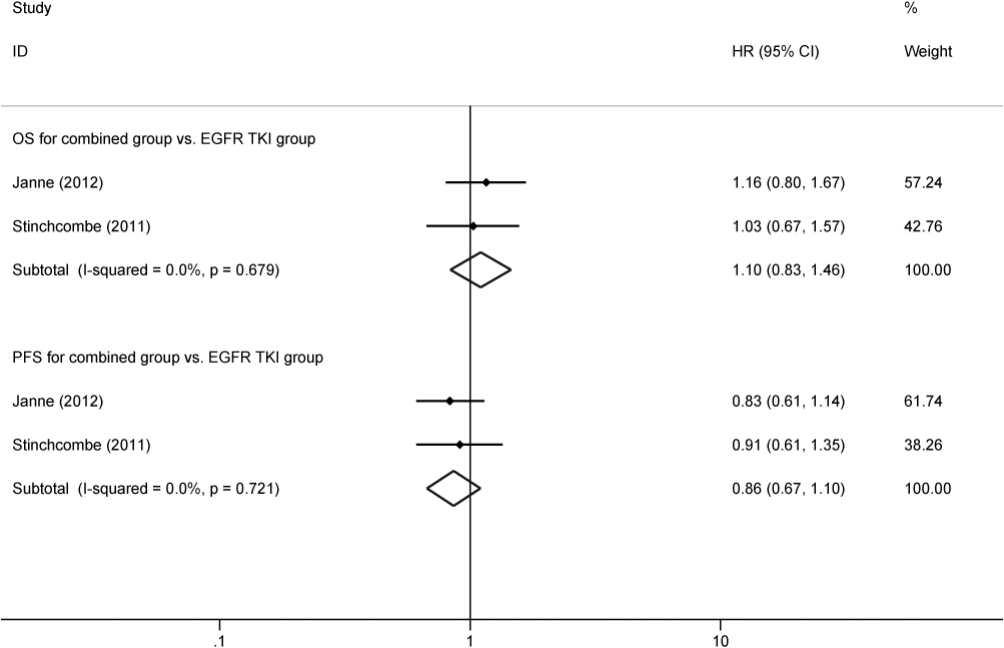
OS and PFS for synchronous combination group vs. EGFR TKIs group.

Two trials [8,13] involving 183 patients reported OS and PFS, comparing the synchronous combination of TKIs and chemotherapy vs. TKIs alone. FEM was applied, as the heterogeneity of the two trials was low (OS: *P* = 0.679, *I* ^*2*^ = 0.0%, PFS: *P* = 0.721, *I* ^*2*^ = 0%). The systematic review showed that there was no significant difference in OS between the two groups (HR = 1.10, 95%CI: 0.83–1.46, *P* = 0.492) (Fig 4). TKIs synchronous, combined with chemotherapy had significantly lower risk of progression, compared with EGFR TKI alone (HR = 0.86, 95%CI: 0.67–1.10, *P* = 0.228) (Fig 4). Due to incomplete data, the systematic review of the ORR comparing the synchronous combination of TKIs and chemotherapy vs. TKIs alone has not been completed.

### Grade 3–4 toxicity analysis

Compared with chemotherapy alone in patients with advanced NSCLC, the patients who received synchronous combination of chemotherapy and EGFR TKIs presented a significant increase in the incidence of grade 3/4 anemia and rash (anemia: RR = 1.40, 95%CI = 1.10–1.79, *P* = 0.007; rash: RR = 7.43, 95%CI = 4.56–12.09, *P*<0.001). There was no difference between the two groups in the incidence of other grade 3/4 toxicity reactions including: leukopenia, neutropenia, thrombocytopenia, fatigue, nausea, vomiting and diarrhea (Table 2). Compared with EGFR TKIs and monotherapy, the patients who received synchronous combination therapy presented with a significant increase in the incidence of grade 3/4 anemia and fatigue (anemia: RR = 6.71, 95%CI = 1.25–35.93, *P* = 0.026; fatigue: RR = 9.60, 95%CI = 2.28–40.86, *P* = 0.002). For neutropenia, thrombocytopenia, rash and diarrhea, the incidence of the two groups were similar (Table 2).

**Table 2:** Grade 3/4 toxic reactions synchronous combined therapy vs. chemotherapy or EGFR TKIs alone.

Toxicity (Grade 3–4)	Trials	Therapy with chemotherapy and TKIs	Therapy with chemotherapy	Heterogeneity *P* value *I* ^*2*^		RR(95%CI)	*P* value
**combined group vs. chemotherapy group**							
leukopenia	4	85/1841	78/1483	0.340	10.70%	0.98 (0.73, 1.32)	0.901
neutropenia	5	215/1884	203/1527	0.976	0.00%	0.95 (0.80, 1.14)	0.580
anemia	5	144/2244	94/1527	0.824	0.00%	1.40 (1.10, 1.79)	0.007
thrombopenia	4	144/1560	111/1186	0.634	0.00%	1.11 (0.88, 1.41)	0.360
fatigue	3	48/850	44/828	0.426	0.00%	1.06 (0.71, 1.58)	0.771
rash	5	187/2244	17/1527	0.171	37.50%	7.43 (4.56, 12.09)	0.000
nausea	4	85/2193	58/1483	0.385	2.00%	1.11 (0.79, 1.56)	0.541
vomiting	4	93/2193	69/1491	0.604	0.00%	1.05 (0.77, 1.44)	0.749
diarrhea	5	246/2244	82/1527	0.000	89.00%	2.14 (0.84, 5.49)	0.112
**combined group vs. EGFR TKI group**							
neutropenia	2	42/151	1/132	0.017	82.60%	8.18(0.08,875.69)	0.378
anemia	2	11/151	1/132	0.799	0.00%	6.71 (1.25, 35.93)	0.026
thrombopenia	2	7/151	1/132	0.415	0.00%	4.46(0.76, 26.28)	0.098
fatigue	2	22/151	2/132	0.483	0.00%	9.60 (2.28, 40.86)	0.002
rash	2	10/151	8/132	0.657	0.00%	1.07 (0.44, 2.63)	0.876
diarrhea	2	10/151	7/132	0.727	0.00%	1.25(0.49, 3.21)	0.644

### Analysis of publication bias

Egger’s test and Begg's test were conducted to evaluate the publication bias of the studies. The summary of the results is shown in Table 3. The results revealed no publication bias.

**Table 3:** Comparison of efficacy between combined therapy and chemotherapy or EGFR TKIs alone.

	Heterogeneity		HR/RR	Begg's test		Egger's test	
	*P* value	*I* ^*2*^	(95%CI)	*Z*	*P*	*t*	*P*
**Synchronous combined group vs. chemotherapy group**							
OS	0.953	0.00	1.05(0.98–1.12)	0.90	0.368	-0.48	0.653
PFS	NE	NE	0.77(0.51–1.17)	NE	NE	NE	NE
TTP	0.920	0.00	0.94(0.89–1.00)	0.75	0.452	-0.91	0.413
ORR	0.475	0.00	1.07(0.98–1.17)	1.22	0.221	2.46	0.091
**Synchronous combined group vs. EGFR TKIs group**							
OS	0.679	0.00	1.10(0.83–1.46)	NE	NE	NE	NE
PFS	0.721	0.00	0.86(0.67–1.10)	NE	NE	NE	NE

## Discussion

In our systematic analysis, synchronous combination therapy of chemotherapy and EGFR TKIs failed to significantly improve OS, TTP and ORR, compared with chemotherapy monotherapy or EGFR TKIs in patients with advance NSCLC. Moreover, synchronous combination therapy increased the incidence of anemia and rash, compared with chemotherapy, and increased the incidence of anemia, thrombocytopenia and fatigue, compared with TKIs. Overall, the results of synchronous combination therapy of chemotherapy and EGFR TKIs are disappointing. TKIs and chemotherapy have different mechanisms; gefitinib and erlotinib are cytostatic, whereas chemotherapy drugs act by cytotoxicity. The anti-tumor effect of TKIs, by arresting the cell cycle, may lower the sensitivity of cytotoxic agents [14–16]. Furthermore, patients are enrolled unselected: patients with EGFR wild-type and patients with EGFR mutations, lung adenocarcinoma and non-adenocarcinoma are all included. The current studies have confirmed that EGFR TKIs had a higher efficacy in patients with lung adenocarcinoma and patients with EGFR mutations. Therefore, the efficacy of synchronous combination therapy needs further stratified study.

Nyati et al summarized that there were at least three potential interaction mechanisms between EGFR inhibitors and chemotherapy: through the cell cycle, through DNA repair and through EGFR signaling [17]. Pre-clinical trials have shown that the schedule of gemcitabine followed by gefitinib might improve efficacy compared to gemcitabine alone, because this schedule resulted in decreased AKT phosphorylation, increased poly(ADP-ribose) polymerase cleavage, and increased tumor cell apoptosis in the treatment of head and neck carcinoma [18]. The results of the systematic analysis were inconsistent with the animal trials of chemotherapy synchronous combined with EGFR TKIs [19,20]. One reason is that the chemotherapeutic drug in animal trials is applied at a lower dose. The chemotherapy drugs applied in the enrolled clinical trials were standard amounts, which might have negated the synergistic effect of EGFR TKIs and chemotherapy. Another possible reason may be derived from tumor implantation in animal models. Compared with orthotropic tumors, subcutaneous ectopic tumor implants failed to interact with the microenvironment of lung cells, which related to the proliferation and the effect to drugs [21,22].

There is scant similar systematic analysis about the combination of chemotherapy and TKIs by the synchronous and interleaved modes. Only Ouyang et al. [23] and Chen et al. [24] conducted two systematic reviews, which merged the two combination modes. Although the systematic analysis showed that combination therapy was superior to chemotherapy, we believe that its main contribution comes from alternating the combination. There was a meta-analysis which compared the efficacy of first-line TKIs (erlotinib or gefitinib) followed by chemotherapy after progression with the reverse treatment in patients with *EGFR*-mutant NSCLC. The result exhibited that the OS of first-line chemotherapy followed upon progression by a TKI was not inferior to that of the inverse sequence in patients with *EGFR*-mutant NSCLC [25]. Another meta-analysis compared the efficacy of chemotherapy plus multitargeted antiangiogenic tyrosine kinase inhibitors (sorafenib, cediranib, vandetanib) with that of chemotherapy alone in advanced NSCLC. The result exhibited that chemotherapy plus multitargeted antiangiogenic TKI was found to significant improve PFS and ORR but not OS compared to chemotherapy alone [26].

EGFR inhibitory therapies are also used against head and neck and colorectal cancer. Similar to NSCLC, gefitinib combined with chemotherapy did not improve outcome compared to chemotherapy alone in head and neck and colorectal cancer [27,28]. In addition to TKI, anti-EGFR monoclonal antibodies (such as Cetuximab) were also often used combined with chemotherapy for NSCLC. A meta-analysis published in The Cochrane Library exhibited that the combination of chemotherapy and cetuximab improved OS compared to chemotherapy alone for the first-line treatment of NSCLC [29]. Bortezomib, that contributed to clinical response of squamous cell carcinoma of the head and neck (SCCHN), antagonized cetuximab- and radiation-induced cytotoxicity, degradation of EGFR, and enhanced pro-survival signal pathway activation in SCCHN tumor biopsies and in the cell line UMSCC-1 [30]. As for clinical data on TKI and radiotherapy, a phase II clinical trial showed that the combination of erlotinib with cisplatin and radiotherapy did not increase complete response rate (CRR) or PFS compared to cisplatin and radiotherapy in patients with locally advanced HNSCC [31].

There are some limitations in this study. First, several enrolled studies have not reported the HR and 95% CI for survival data. The small sample size of the clinical trials, compared with the combination group with EGFR TKI group, meant that the data included in the systematic review was limited. Third, the studies did not report the data of patients with EGFR mutations, EGFR wild-type, adenocarcinoma and squamous cell carcinoma. In conclusion, we found that the synchronous combination of chemotherapy and TKIs did not obtain satisfactory results. To obtain more convincing data, rigorous phase III clinical trials are needed to further explore the potential benefits of the efficacy of chemotherapy combined with TKIs in advanced NSCLC patients.

## Supporting Information

S1 Fig The biases of the studies assessed with the criterion recommended by Cochrane. (A) Risk of bias graph. (B) Risk of bias summary.
(TIF)

S1 Table PRISMA Checklist. (DOC)
