# Supplementary figures and images for: The Efficacy of Synchronous Combination of Chemotherapy and EGFR TKIs for the First-Line Treatment of NSCLC: A Systematic Analysis
Source: PLoS One. 2015 Aug 18;10(8):e0135829. doi: 10.1371/journal.pone.0135829 (PMC4540576; doi:10.1371/journal.pone.0135829)

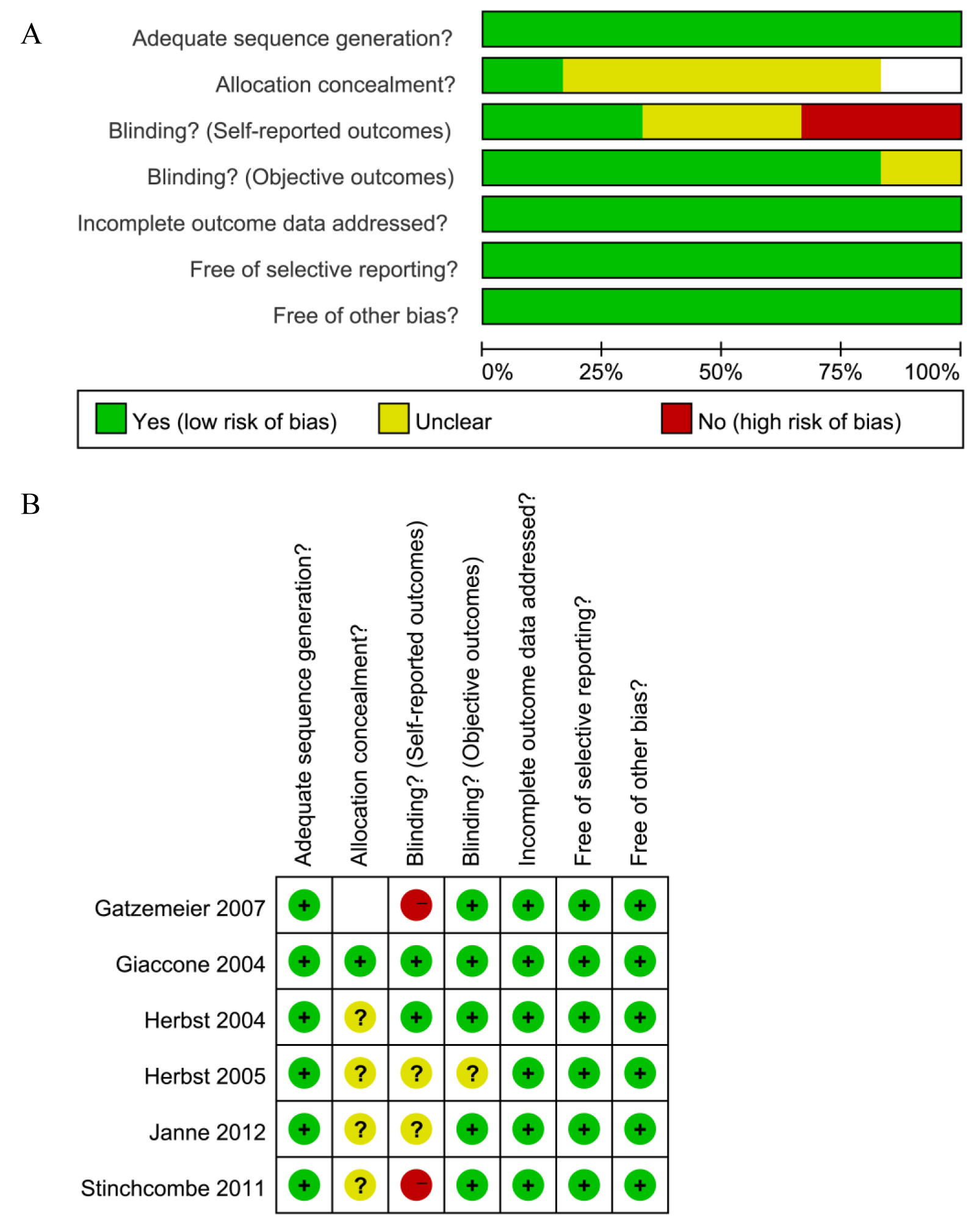

Supplement: S1 Fig — (A) Risk of bias graph. (B) Risk of bias summary. (TIF) [file pone.0135829.s001.tif]
